# Critical aspiration pneumonia during induction of anesthesia in elective abdominal surgery: a case report

**DOI:** 10.1186/s40981-022-00549-w

**Published:** 2022-08-05

**Authors:** Midori Mogami, Yuki Yamazaki, Chiaki Nemoto, Mariko Muto, Youichi Tanaka, Satoki Inoue

**Affiliations:** 1 Department of Anesthesiology, Ohara General Hospital, 6-1 Ohomachi, Fukushima, 960-8611 Japan; 2 Department of Anesthesiology, Fukushima Medical University, 1 Hikarigaoka, Fukushima, Fukushima 960-1295 Japan

**Keywords:** Aspiration pneumonia, Gastrointestinal obstruction, Fasting period, Point-of-care gastric ultrasonography

## Abstract

**Background:**

We experienced the critical aspiration pneumonia during induction of anesthesia in elective abdominal surgery which standard fasting period was complied with.

**Case presentation:**

A 64-year-old male was scheduled for gastrojejunostomy because of gastrointestinal obstruction. He fasted from the night before surgery. General anesthesia was induced, and cricoid pressure was applied during intubation. However, he vomited huge amount of gastric contents. The scheduled surgery was performed without surgical complications, and postoperatively respiratory management, including mechanical ventilation with prone positioning, was performed in high care unit. He was extubated on postoperative day 2. He was discharged from the hospital on POD 25.

**Conclusion:**

The standard fasting period can prevent aspiration pneumonia in most cases. However, even in elective cases without abdominal symptoms, we consider that massive-volume gastric residual contents, especially in susceptible cases. We suggest that point-of-care gastric ultrasonography be performed in suspicious cases before induction of anesthesia.

## Background

Gastric regurgitation upon induction of general anesthesia is rare but can induce critical aspiration pneumonia, which is one of the major causes of anesthesia-related death [1, 2]. However, gastric regurgitation in most cases is preventable simply by overnight fasting (> 6 h) in elective surgeries, which is considered to sufficiently reduce gastric residual volume [3]. We experienced a case of critical aspiration pneumonia during induction of anesthesia in elective abdominal surgery.

## Case presentation

A 64-year-old male (167 cm tall, weighing 52 kg) was scheduled for gastrojejunostomy because of gastrointestinal obstruction owing to pancreatic cancer. He could not ingest solids but took liquid nutrition without problems before admission. Preoperative examination indicated slight hypoproteinemia and renal dysfunction (total protein: 6.1 g/dl, albumin: 3.9 g/dl, estimated glomerular filtration rate: 46.5 ml/min/1.73 m^2^). He was admitted to our hospital on the weekend, and intravenous rehydration was performed for 3 days. He was permitted to take oral rehydration until the night before surgery. A nasogastric tube for evacuating the gastric contents was not used before induction of general anesthesia because the patient had no abdominal complaints. General anesthesia was induced with a target-controlled infusion of propofol 3.5 mcg/ml and remifentanil 0.3 mcg/kg/min. Soon after loss of consciousness, 50 mg of rocuronium was administered, and cricoid pressure was applied. Suddenly, the patient vomited a massive volume of gastric contents. While suctioning the vomitus, intubation was achieved using a direct laryngoscope. During intubation, we did not observe a pharyngeal reflex or a laryngeal reflex. Six liters of gastric contents were evacuated via the nasogastric tube. With suction via the tracheal tube, we were able to drain the aspirated gastric contents. At this point, we considered postponing the operation; however, considering the patient’s prognosis for the primary disease and that the operation time was not anticipated to be long, we discussed the options with the surgeon and decided to perform gastrojejunostomy.

**Fig. 1:**
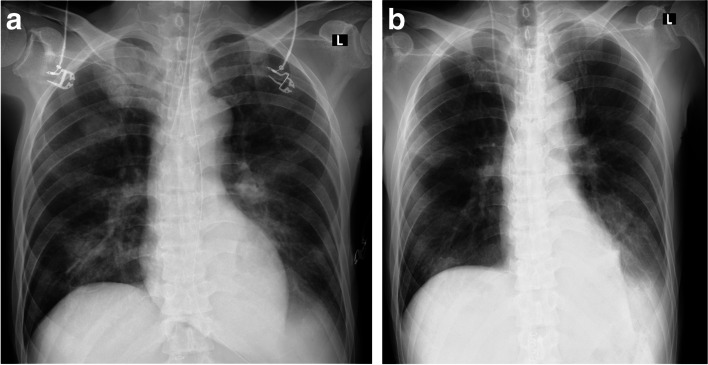
Opacities in the right lower lung lobe were found immediately after surgery. Chest radiograph on POD 10. Dullness at the left costophrenic angles remained, but the opacities in the right lower lung lobe were improving after treatment. POD, postoperative day

Gastrojejunostomy was completed without surgical complications, and the patient was admitted to the high care unit for respiratory management postoperatively, including mechanical ventilation with prone positioning. A chest radiograph obtained at admission to the high care unit is shown in Fig. 1a. Arterial blood gases were measured after admission to the high care unit after initiating mechanical ventilation using synchronized intermittent mandatory ventilation with the following settings: fraction of inspired oxygen (FIO_2_): 0.7, positive end-expiratory pressure: 8 cm H2O, pressure support: 5 cm H2O, tidal volume: 400 ml, and respiratory rate: 12 breaths/min. Blood gas results were as follows: pH: 7.205, partial pressure of arterial oxygen: 91.6 mmHg, partial pressure of arterial carbon dioxide: 74.1 mmHg, and bicarbonate: 29.3 mmol/L. Prone positioning was performed for approximately 4 h on the first day after the surgery. Additionally, a huge volume of sputum drained during the prone positioning, and oxygenation improved drastically the next day. The partial pressure of arterial oxygen/FIO_2_ ratio was 381 mmHg with an FIO_2_ of 0.7 and positive end-expiratory pressure of 8 cmH2O.

Tazobactam/piperacillin 13.5 g/day, sivelestat 250 mg/day, and methylprednisolone 1000 mg/day were administered. Both the patient’s respiratory status and his hemodynamic status were difficult to manage; however, the hemodynamics were responsive to treatment with noradrenaline and vasopressin and fluid resuscitation. He was extubated on postoperative day (POD) 2. His general condition gradually improved, and he was discharged from the hospital on POD 25. A chest radiograph obtained on POD 10 is shown in Fig. 1b.

## Discussion

Aspiration pneumonia upon induction of general anesthesia is a chemical pneumonia, which can develop into critical illness [1]. In our case, we did not anticipate that there would be 6 L of gastric remnant contents. Even though gastrointestinal obstruction was present, the patient was able and permitted to take liquid nutrition before and after admission. Optimistically, we thought that clear water could pass through the narrow segmental lesion. Retrospectively, reviewing our case, we should have inserted a nasogastric tube before induction of general anesthesia. We expected a certain amount of gastric contents; therefore, we applied cricoid pressure at the induction of anesthesia. However, in cases of massive vomiting, as in this case, it is very difficult to prevent aspiration using only cricoid pressure. Cricoid pressure has been used for possible full stomach cases; however, there is a lack of scientific evidence indicating a benefit to prevent aspiration pneumonia and possible complications [4]. Actually, it has been suggested that cricoid pressure could be very dangerous in some cases, such as in our case, because massive vomiting under cricoid pressure may provoke esophageal rupture [5].

**Fig. 2:**
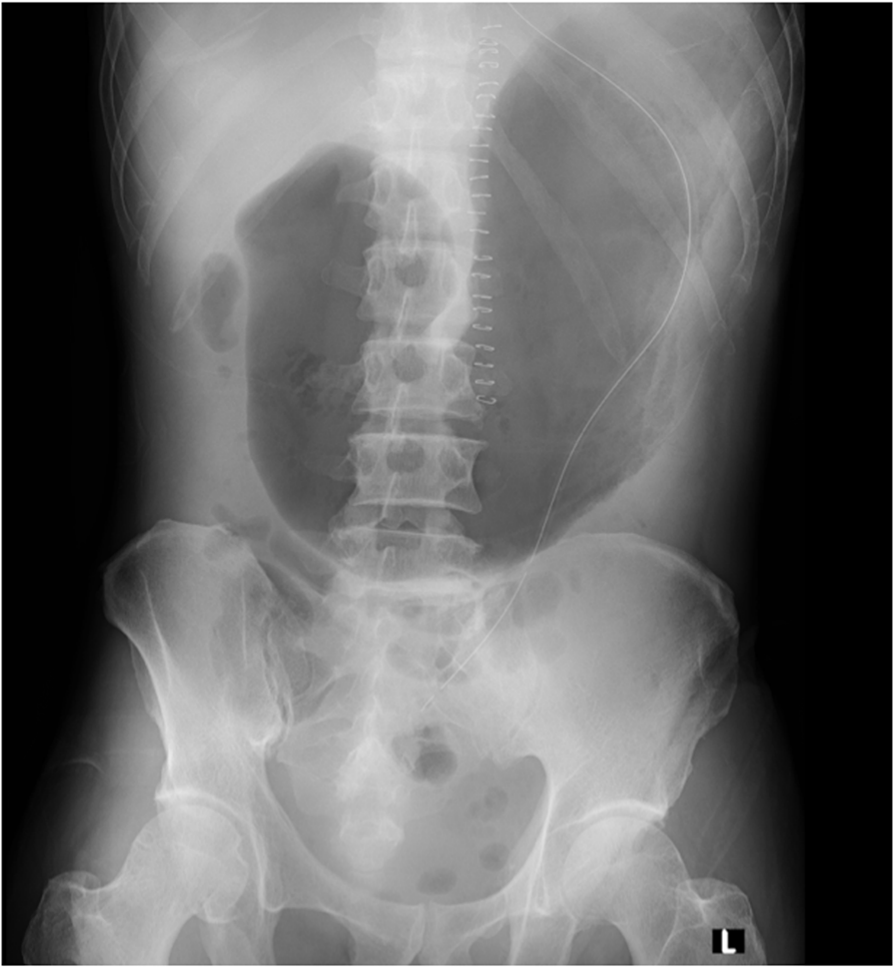
Abdominal radiograph on POD 10 showing the patient’s distended stomach, which was likely owing to aerophagia and nasogastric tube malfunction. The tube was repositioned to drain the air. POD, postoperative day

Preoperative X-ray and computed tomography imaging did not indicate a distended stomach and complete obstruction of the gastrointestinal tract. However, radiography on POD 10 showed the patient’s distended stomach, which was likely a result of aerophagia and nasogastric tube malfunction (Fig. 2). Because of the gastrointestinal obstruction owing to pancreatic cancer, the patient’s stomach had gradually distended to the size it was at the time of surgery because of prolonged oral intake. Therefore, we should have considered a possible critically full stomach even though a standard fasting period was complied with, and abdominal symptoms were absent. Recently, point-of-care gastric ultrasonography has been reported to be promising for estimating the presence of gastric contents [6]. This assessment should have detected the huge gastric contents in our case.

Prone ventilation therapy was performed immediately after the operation in our case. Physicians may hesitate to implement the prone position soon after open abdominal surgery; however, this positioning is worth using without hesitation because there is a growing body of evidence stating that the prone position could be beneficial for respiratory management [7]. Drainage of aspirated contents contributes to infection source control. Sivelestat and methylprednisolone were used in this case. These medications may be therapeutic options; however, consensus regarding the use of these drugs in aspiration pneumonia is lacking.

In conclusion, aspiration pneumonia that occurs at the induction of anesthesia is very rare but critical once it happens and may require aggressive intensive care. The standard fasting period can prevent this event in most elective surgical cases. However, even in elective cases with no abdominal symptoms, we must consider that huge gastric residual contents, which may be several times the volume of the standard stomach volume, can be present, especially in susceptible cases. We suggest that point-of-care gastric ultrasonography be performed in suspicious cases before induction of anesthesia.

## Data Availability

Not applicable

## Abbreviations

POD: Postoperative day
FIO_2_: Fraction of inspired oxygen
